# Digital health literacy and its behavioral correlates among medical undergraduates in central China: a cross-sectional study

**DOI:** 10.3389/fpubh.2026.1880430

**Published:** 2026-07-09

**Authors:** Yu Wang, Jiamin Wang, Bin Wei, Ying Wang, Zhengrong Zhou, Zhuoyun Wang

**Affiliations:** 1 Department of Hospital Management Teaching and Research Section, The Second Affiliated Hospital of Anhui Medical University, Hefei, China; 2 School of Health Management, Anhui Medical University, Hefei, China; 3 Department of Network and Information Technology Management, The Second Affiliated Hospital of Anhui Medical University, Hefei, China

**Keywords:** cross-sectional study, digital health literacy, eHEALS, health information behaviors, medical undergraduate students

## Abstract

**Background:**

Digital health literacy (DHL) is a core competency for future healthcare workers, yet its predictors determinants among medical undergraduates in Central China remain unclear.

**Methods:**

A cross-sectional study was conducted among 1,130 medical undergraduates in Anhui Province using stratified cluster sampling. DHL was assessed using the validated Chinese version of the eHealth Literacy Scale (eHEALS). Hierarchical multiple linear regression was used to identify independent predictors of DHL.

**Results:**

The mean DHL score was 29.78 ± 5.57, indicating a moderate level. After adjusting for confounders, health information retrieval efficiency, authoritative source identification ability, and reliance on professional medical databases were the strongest positive predictors of DHL. Sociodemographic factors showed no significant independent association with DHL.

**Conclusions:**

DHL among medical undergraduates is primarily predicted by modifiable behavioral factors rather than innate sociodemographic backgrounds. Universal competency-oriented training programs are needed to improve DHL in this critical public health workforce.

## 1 Introduction

Against the backdrop of China's “Internet + Medical Health” national strategy, digital technologies have fundamentally transformed healthcare delivery and health information ecosystems (1). While this digitization has expanded access to medical resources, it has also created unprecedented challenges: the exponential growth of heterogeneous online health information (2), coupled with the rampant spread of misleading medical content and unfounded health rumors (3, 4), has significantly increased risks for both healthcare providers and the general public (5). In this context, digital health literacy (DHL)—the ability to efficiently access, critically evaluate, and appropriately apply digital health information (6, 7)—has evolved from a supplementary skill to a core professional competency for all healthcare practitioners (8–10). As the future backbone of China's healthcare system (11, 12), medical undergraduates are in a critical period of professional identity formation and competency development (13–15). Their DHL not only shapes their personal lifelong learning capabilities and clinical decision-making quality but also determines their effectiveness as health educators and communicators who will deliver evidence-based health information to the public (12, 16). Therefore, investigating DHL status and identifying evidence-based improvement pathways among medical students is a critical priority for advancing higher medical education reform (17) and ensuring the high-quality development of the future public health workforce (18, 19).

DHL was first conceptualized by Norman and Skinner in 2006 (6) as a specialized form of literacy that integrates foundational digital skills, health knowledge, and critical thinking abilities. It is defined as the comprehensive capacity of individuals to effectively navigate electronic health resources to address health-related problems, encompassing three interrelated dimensions: foundational literacy, health literacy, and information literacy (20). Unlike general digital literacy, which focuses on broad technology proficiency, DHL is specifically tailored to healthcare scenarios and medical professional contexts, representing an indispensable professional extension of health literacy in the digital age (21).

Regarding the global research landscape, the pooled mean eHEALS score among medical students worldwide is 28.6 (95% CI: 26.3–30.9) (8). In China, medical students' scores generally range from 26 to 31, indicating an overall moderate level (18). A consistent pattern of “adequate health awareness but weak higher-order skills” has been observed, with particularly notable deficiencies in critical evaluation of health information and multi-source verification capabilities (11). In some regions, the proficiency rate for these advanced skills is less than 40% (12). Concerning influencing factors, existing research has converged on two dominant perspectives: one emphasizes the deterministic role of sociodemographic factors such as gender, household registration, and family background (22), while the other argues that modifiable digital usage behaviors and health information processing abilities play a more critical role in literacy development (23). Compared with immutable sociodemographic characteristics, behavioral and ability-related factors can be optimized through curricular interventions and training programs, making them a focal point of current DHL research (9). In this study, we collectively refer to these modifiable behavioral and ability-related factors as “behavioral determinants,” defined as digital practice behaviors and information processing skills that can be shaped through educational interventions and significantly predict individuals' DHL levels. Specifically, this concept includes two dimensions: health-related digital resource usage behaviors (e.g., frequency of using medical learning websites, health-related apps, and reliance on professional databases) and health information processing abilities (e.g., information retrieval efficiency, ability to identify authoritative sources, medical knowledge judgment skills, and proactive multi-channel verification behaviors). These two categories of factors are the core modifiable predictors of medical students' DHL (12).

Despite extensive research examining the individual effects of digital usage behaviors or information processing abilities on DHL, three critical gaps remain in the literature. First, few studies have systematically integrated both categories of behavioral determinants into a single analytical framework, making it difficult to compare their relative predictive contributions (8). Second, the independent predictive effect of health-related digital usage behaviors on DHL remains controversial, as previous studies have often failed to simultaneously control for sociodemographic factors and information processing abilities (24). Third, it remains unclear whether sociodemographic factors retain independent effects after accounting for behavioral and ability variables, which has direct implications for the equitable design of DHL educational interventions (13). Furthermore, there is a paucity of localized empirical studies focusing on medical students in central China. Anhui Province, located in central China, exhibits regional representativeness in terms of economic development level and digital healthcare construction. However, no systematic study has yet investigated the behavioral predictors of DHL among undergraduate medical students in this region.

To address these gaps, this study is grounded in the reality of medical education in central China. We conducted a cross-sectional survey among undergraduate medical students at a medical university in Anhui Province using the validated Chinese version of the eHEALS scale and a self-designed questionnaire on health information behaviors and abilities. The study aims to systematically analyze the current status of DHL in this population, comprehensively explore the independent predictive effects of the two major behavioral predictors (health-related digital usage behaviors and health information processing abilities) on DHL, and further examine the residual independent effects of sociodemographic factors after controlling for these variables. Accordingly, we propose the following three research hypotheses: (1) Health-related digital usage behaviors, health information processing abilities, and DHL are all significantly positively correlated among medical students; (2) Health information retrieval efficiency, ability to identify authoritative sources, medical knowledge judgment skills, multi-channel information verification behaviors, and standardized health-related digital usage behaviors are all positive independent predictors of medical students' DHL; (3) After controlling for health-related digital usage behaviors and health information processing abilities, sociodemographic factors have no significant independent effects on medical students' DHL. The findings of this study will not only enrich the localized research evidence on medical students' DHL in Central China but also provide evidence-based guidance for public health departments and medical schools to develop targeted DHL training programs, ultimately improving the digital health competency of the future health workforce.

## 2 Methods

### 2.1 Study design and setting

This cross-sectional study was conducted at the largest medical college in Anhui Province, Central China, between May and June 2026. A cross-sectional design was selected as it is an efficient and cost-effective approach to assess the prevalence of health-related outcomes and their associated factors in large populations at a single time point (25). This design is particularly suitable for exploratory studies aimed at identifying potential predictors of DHL (26), which was the primary objective of this research.

### 2.2 Participants and sampling procedure

A stratified cluster sampling method was used to recruit participants. Stratification was performed based on academic year (first to fifth year) and major (clinical medicine, medical technology, public health, health management, etc.) to ensure proportional representation of all subgroups in the undergraduate medical student population. This sampling approach was chosen for three reasons. First, stratified sampling ensures that key subgroups—particularly different academic years and majors—are adequately represented, which enhances the representativeness of the sample and improves the external validity of the findings. Second, cluster sampling (with classes as clusters) is the most practical method for large-scale surveys in educational settings, where students are naturally organized into classes and individual random sampling would be logistically prohibitive and disruptive to the educational environment (27). Third, the combination of stratification and clustering balances the methodological rigor of stratified sampling with the operational feasibility of cluster sampling, making it the optimal design for school-based survey research (28, 29). Classes were taken as the cluster sampling unit, and all eligible students in selected classes were invited to participate.

#### 2.2.1 Inclusion and exclusion criteria

Full-time undergraduate medical students who were officially registered at the university and present in class on the survey day were included. These inclusion criteria ensured that all participants were actively engaged in the standard medical education program and could complete the survey under standardized conditions, thereby guaranteeing the homogeneity of the study population (30).

Exchange students, graduate students, and students in associate-to-bachelor's degree programs were excluded. These exclusion criteria were established to focus exclusively on students receiving a unified 5-year general undergraduate medical education, which is the target population for DHL training interventions in medical schools. Students in these excluded categories have systematically different curricula, training pathways, and access to digital resources compared with regular 5-year undergraduate medical students; including them would introduce uncontrolled confounding factors that could compromise the validity of the findings (30).

All students who met the criteria were invited to participate during the summary sessions held at the end of the academic year. Researchers were present at the beginning of each session, explained the content of informed consent, and guided participants to complete the electronic questionnaire via QR code scanning using their personal smartphones. Participation was completely voluntary, and participants could withdraw from the survey at any time without negative consequences. All questionnaires were completed independently in the classroom, and responses were automatically submitted to a secure encrypted online platform.

A total of 1,141 students were invited on-site, and 1,130 completed the electronic questionnaires, with an effective response rate of 99%. The sample size was calculated using the single-sample mean formula for cross-sectional studies. Based on a pooled mean eHEALS score of 28.6 (SD = 5.5) from a previous meta-analysis (6), a two-sided significance level of α = 0.05, and statistical power of 80%, the minimum required sample size was 384. Considering a design effect of 1.5 for stratified cluster sampling and a 10% expected non-response rate, the target sample size was adjusted to 634. The final sample size of 1,130 was more than sufficient to detect significant associations in this study.

### 2.3 Ethical considerations

This study followed the principles of the 2023 updated Declaration of Helsinki (31, 32). Ethical approval was obtained from the Ethics Committee of the Second Affiliated Hospital of Anhui Medical University (Approval No. SL-YX2026-097). All participants provided electronic informed consent prior to data collection (29). Participation was entirely voluntary, and participants could withdraw at any time without penalty. No personal identifying information was collected, and all responses were anonymized and stored on a secure encrypted server accessible only to the research team. The raw data will be retained for 5 years in accordance with Chinese research data management regulations and then securely deleted.

### 2.4 Survey tools

#### 2.4.1 Demographic characteristics

Social-demographic information of participants were collected, including the following variables: gender, age, family Residence (urban/township/rural), ancestral hometown (within province/outside the province), major, academic year, scholarship recipient status (yes/no), only child status (yes/no), father's highest educational attainment, mother's highest educational attainment, and self-assessed family economic status (rated as extremely difficult/comparatively difficult/moderate/fairly comfortable/very comfortable).

#### 2.4.2 The Chinese version of eHEALS

This scale was developed by Norman and Skinner in 2006 (6) to assess individuals' core competencies in acquiring, evaluating, and applying digital health information. The original version features a single-dimensional, 8-item structure using a five-point Likert scale (1 = Strongly Disagree, 5 = Strongly Agree), with total scores ranging from 8 to 40 points. Higher scores indicate stronger DHL. This study used the Chinese version translated and revised by Guo et al. (33), which demonstrated a Cronbach's α coefficient of 0.913 in the original validation study, indicating good reliability. In the present study sample, the scale yielded a Cronbach's α coefficient of 0.962, with item-total correlations ranging from 0.803 to 0.871, indicating excellent internal consistency reliability.

#### 2.4.3 Health information behavior and competence questionnaire

This questionnaire was used to collect data on participants' health status, health-related digital use practices, and health information processing behaviors and competencies (34). It was developed based on a systematic review of existing literature on digital health literacy and health information behaviors, and adapted specifically for the Chinese medical student context.

The questionnaire was developed through a systematic literature review (34), followed by expert panel review for content validity. A panel of experts in public health, clinical medicine, health informatics, and biostatistics evaluated each item on relevance, clarity, comprehensiveness, and appropriateness using a four-point Likert scale. Item-level content validity indices (I-CVI) were calculated, and items with I-CVI below 0.78 were revised or removed. The scale-level content validity index (S-CVI/Ave) was 0.92, indicating excellent content validity. Based on expert feedback, several items were reworded for improved clarity and cultural appropriateness, and redundant items were removed. The final questionnaire demonstrated acceptable internal consistency (Cronbach's α = 0.742 for the four-item competency dimension).

It consists of two parts, with a total of 14 core variables measured through five-point Likert scales, frequency ranking options, and categorical selection questions. The first part focuses on health status and health-related digital use practices, investigating respondents' health background and digital use practices in health and medical learning scenarios, covering overall self-rated health, presence of chronic conditions requiring long-term management, daily internet usage duration, types of digital devices commonly used for health information access and medical learning, frequency of medical learning website usage, daily internet access methods, and prior training experience in information retrieval or evidence-based medicine. The second part assesses health information behaviors and competencies, evaluating respondents' practical behaviors and core cognitive skills in digital health information processing, including frequency of health information searches in the past 6 months, frequency of health-related application usage, self-rated efficiency in health information retrieval, self-rated ability to distinguish authoritative sources of health information, self-rated capacity for medical knowledge judgment and health misinformation identification, tendency of proactive multi-channel verification for health information, and primary sources of health information in daily life.

Although the health information processing variables and eHEALS dimensions share conceptual proximity, they measure distinct constructs. Rooted in Bandura's self-efficacy theory, the eHEALS primarily assesses individuals' subjective beliefs and perceived self-efficacy regarding digital health information processing, adopting general descriptive items to evaluate overall competence. In contrast, our health information behavior and competence questionnaire is developed based on information behavior theory, focusing on respondents' practical behaviors and context-specific capabilities reflected in real scenarios, and all items are designed around specific daily usage experiences rather than general self-perception. This distinction is supported by previous research (35), which has shown that perceived eHealth literacy and actual performance on digital health tasks are only moderately correlated (*r* = 0.32) and represent separate constructs.

Reliability testing was conducted on the four five-point Likert self-assessment items that constitute the health information processing competency dimension, including retrieval efficiency, authoritative source identification, medical knowledge judgment, and multi-channel verification tendency. The Cronbach's α coefficient for this dimension was 0.742, indicating strong internal consistency. Health information sources were assessed using a multiple-choice question with seven options (professional databases, search engines, social media, online video platforms, school curriculum/teacher recommendations, peer communication, and other). Respondents could select all applicable sources they used in daily life. Each source was analyzed as an independent binary variable (used vs. not used) in subsequent statistical analyses.

### 2.5 Statistical analysis

Statistical analysis was performed using SPSS 27.0 software (IBM SPSS Statistics for Windows, Version 27.0. Armonk, NY: IBM Corp.). Count data were described as frequency (*n*) and percentage (%). Continuous data were expressed as mean±standard deviation (x ± s). The skewness test was used to assess distribution patterns (a skewness coefficient ≤ 2 was considered to indicate an approximately normal distribution).

Univariate analyses were conducted to assess differences in total DHL scores across categorical variables. Independent samples *t*-tests and one-way analysis of variance (ANOVA) were used for variables with two and more than two groups, respectively; the Brown-Forsythe test was applied when the assumption of homogeneity of variance was violated. Bonferroni correction was applied to adjust for multiple comparisons when analyzing the seven independent binary variables derived from the health information sources question, with an adjusted significance level of α = 0.007.

Further hierarchical multiple linear regression analysis was conducted to examine influencing factors, using total DHL scores as the dependent variable. Consistent with conventional analytical specifications for observational cross-sectional studies, only variables showing statistical significance in univariate analysis were selected as candidate independent variables. This screening rule is adopted to streamline the model structure, reduce the number of irrelevant predictors, effectively mitigate multicollinearity risks, and improve the stability and interpretability of regression results. Variables with *P* < 0.05 in the univariate analysis were selected as candidate independent variables to sequentially construct three progressive models. This stepwise variable selection approach is a standard and widely accepted method in observational studies, recommended by leading biostatistics textbooks and extensively used in previous digital health literacy research (36). It helps identify the most parsimonious set of independent predictors while minimizing the risk of overfitting, which is particularly appropriate given our large sample size of 1,130 participants.

Model I included only sociodemographic variables; Model II added health status and health-related digital use behaviors variables to Model I; Model III further incorporated health information behavior and capability variables. Model fit and residual autocorrelation were assessed using the coefficient of determination (*R*^2^), adjusted coefficient of determination (Adjusted *R*^2^), and Durbin-Watson statistic (37). Multicollinearity among independent variables was evaluated using the variance inflation factor (VIF). A VIF value < 2.5 was considered to indicate no significant multicollinearity. In this study, all VIF values ranged from 1.02 to 2.18, confirming the absence of problematic multicollinearity.

Notably, this study adopted stratified cluster sampling with classes as clusters, but conventional regression models without cluster adjustment were used in inferential statistics. This may lead to slightly underestimated standard errors and inflated statistical test results. All statistical tests in this study were two-tailed, with *P* < 0.05 indicating statistically significant differences. The full questionnaire used for data collection is provided as Supplementary material S1.

## 3 Result

### 3.1 Distribution of DHL scores

The mean total DHL score of the included medical undergraduates was 29.78 ± 5.57, with scores ranging from 8 to 40, indicating an overall moderate level of DHL among the study population. The skewness test showed that DHL scores followed an approximately normal distribution (skewness coefficient ≤ 2), which supported the use of parametric tests in subsequent statistical analyses.

### 3.2 DHL scores by demographic characteristics

The final sample consisted of 529 males (46.8%) and 601 females (53.2%); 438 students (38.8%) were from urban areas, 265 (23.5%) from township areas, and 427 (37.8%) from rural areas; 1,009 students (89.3%) were native to Anhui Province, and 121 (10.7%) were from other provinces; 353 students (31.2%) were only children, and 777 (68.8%) were non-only children; 851 students (75.3%) majored in clinical medicine, 143 (12.7%) in medical technology, 52 (4.6%) in health management, and 84 (7.3%) in other majors; 837 students (74.1%) were in junior grades (first to third year), and 293 (25.9%) were in senior grades (fourth to fifth year).

DHL scores showed significant differences among most demographic groups (Table 1). In particular, DHL scores presented statistically significant differences in relation to gender, family residence, ancestral hometown, only child status, paternal highest educational attainment, maternal highest educational attainment, and self-assessed family economic status. For example, significant differences were found in the total DHL score based on family residence (*F* = 11.696, *P* < 0.001), paternal highest educational attainment (*F* = 6.813, *P* < 0.001), and self-assessed family economic status (*F* = 12.145, *P* < 0.001). Conversely, no significant differences in DHL scores were observed across age groups, field of study, academic year, or scholarship recipient status (all *P* > 0.05).Notably, there was also no statistically significant difference in DHL scores across academic years (first to fifth year, *F* = 0.872, *P* = 0.481), indicating that students' digital health literacy levels did not improve progressively as they advanced through their undergraduate medical training. This unexpected finding carries particularly important educational implications.

**Table 1:** Distribution of DHL scores according to demographic characteristic groups.

Item		Frequency	Percentage (%)	Digital health literacy score	*F*/*t*-value	*P*-value
Gender	Male	529	46.8	30.31 ± 5.816	1.565	0.002
	Female	601	53.2	29.31 ± 5.297		
Age (years)	≤ 18	535	47.3	29.92 ± 5.566	0.336	0.715
	19	317	28.1	29.68 ± 5.49		
	≥20	278	24.6	29.61 ± 5.664		
Family residence	Urban	438	38.8	30.77 ± 5.608	11.696	< 0.001
	Township	265	23.5	29.28 ± 5.390		
	Rural	427	37.8	29.07 ± 5.492		
Ancestral hometown	Within province	1,009	89.3	29.64 ± 5.491	−2.342	0.019
	Outside the province	121	10.7	30.89 ± 6.065		
Field of study	Clinical	851	75.3	29.97 ± 5.667	2.127	0.075
	Medical technology	143	12.7	29.72 ± 5.133		
	Health management	52	4.6	29.00 ± 5.466		
	Other	84	7.3	28.42 ± 5.121		
Academic year	Junior grade	837	74.1	29.86 ± 5.546	0.819	0.413
	Senior grade	293	25.9	29.55 ± 5.625		
Scholarship status	No	884	78.2	29.94 ± 5.609	1.889	0.059
	Yes	246	21.8	29.18 ± 5.378
Only child	No	777	68.8	29.37 ± 5.502	−3.610	< 0.001
	Yes	353	31.2	30.66 ± 5.612		
Father's highest education level	Elementary school or below	96	8.5	27.46 ± 5.466	6.813	< 0.001
	Junior high School	405	35.8	29.22 ± 5.119		
	High school/vocational school	274	24.2	30.28 ± 5.82		
	Junior college	125	11.1	30.14 ± 5.349		
	Bachelor's degree	198	17.5	30.82 ± 5.85		
	Graduate and above	32	2.8	31.41 ± 5.645		
Mother's highest education level	Elementary school or below	211	18.7	28.72 ± 5.273	5.759	< 0.001
	Junior high school	383	33.9	29.21 ± 5.513		
	High school/vocational school	253	22.4	30.11 ± 5.54		
	Junior college	109	9.6	30.56 ± 4.594		
	Bachelor's Degree	151	13.4	31.33 ± 6.352		
	Graduate degree and above	23	2	31.3 ± 5.182		
Self-reported household economic status	Extremely difficult	44	3.9	30.14 ± 6.656	12.145	< 0.001
	Comparatively difficult	213	18.8	28.07 ± 5.182		
	Moderate	717	63.5	29.76 ± 5.59		
	Fairly comfortable	148	13.1	32.02 ± 4.756		
	Very comfortable	8	0.7	32.88 ± 5.693		

### 3.3 Univariate analysis of DHL by health status, health-related digital use behaviors and health information behaviors

Univariate analyses were performed to examine differences in total DHL scores across health status, health-related digital use behaviors characteristics (Table 2), as well as health information behaviors and competencies (Table 3).

**Table 2:** Univariate analysis of DHL scores by health status and health-related digital use behaviors characteristics.

Variable category and item	Grouping/option	Frequency	Percentage (%)	Digital health literacy score	*F*/*t*-value	*P*-value
Self-rated health	Poor	10	0.9	25.70 ± 10.520	46.077	< 0.001
	Fair	40	3.5	26.88 ± 6.086		
	Good	422	37.3	27.82 ± 5.131		
	Very Good	462	40.9	30.33 ± 4.669		
	Excellent	196	17.3	33.46 ± 5.764		
Chronic disease requiring long-term management	No	1,084	95.9	29.80 ± 5.617	0.640	0.522
	Yes	46	4.1	29.26 ± 4.203		
Daily internet usage time (h)	< 2	30	2.7	31.13 ± 7.528	2.575	0.036
	2–4	303	26.8	30.46 ± 5.315		
	4–6	435	38.5	29.64 ± 5.508		
	6–8	218	19.3	29.11 ± 5.435		
	>8	144	12.7	29.45 ± 5.858		
Common digital devices						
Smartphone	Not used	17	1.5	30.29 ± 6.469	0.387	0.699
	Usage	1,113	98.5	29.77 ± 5.554		
Laptop/Tablet	Not used	308	27.3	28.97 ± 5.786	−2.972	0.003
	Usage	822	72.7	30.08 ± 5.454		
Desktop computer	Not in use	1,028	91.0	29.73 ± 5.525	−0.894	0.372
	Usage	102	9.0	30.25 ± 5.966		
Other (smartwatches, etc.)	Not used	1,014	89.7	29.61 ± 5.55	−3.006	0.003
	Usage	116	10.3	31.24 ± 5.51		
Frequency of medical learning network usage	Multiple times daily	246	21.8	31.04 ± 5.828	13.375	< 0.001
	Once daily	160	14.2	29.84 ± 5.402		
	Several times per week	421	37.3	30.11 ± 4.996		
	Several times per month	162	14.3	29.39 ± 5.629		
	Rarely or never	141	12.5	26.95 ± 5.881		
Daily internet type						
Campus WiFi	Not Used	126	11.2	29.58 ± 5.404	−0.419	0.675
	Usage	1,004	88.8	29.8 ± 5.588		
Monthly average mobile data usage (GB)	Not used	149	13.2	29.16 ± 5.726	1.483	0.217
	≤ 10	209	18.5	30.35 ± 5.76		
	11–30	363	32.1	29.61 ± 5.169		
	≥31	409	36.2	29.85 ± 5.734		
Public WiFi	Not used	1,056	93.5	29.77 ± 5.552	−0.165	0.869
	Usage	74	6.5	29.88 ± 5.793		
Information retrieval/ evidence-based medicine training	Systematically studied	193	17.1	31.37 ± 5.418	15.258	< 0.001
	Briefly familiar with	477	42.2	30.03 ± 5.377		
	Never studied	460	40.7	28.84 ± 5.65		

**Table 3:** Univariate analysis of DHL scores by health information behaviors and competencies.

Variable category and item	Group/option	Frequency	Percentage (%)	Digital health literacy score	*F*/*t*-value	*P*-value
Health information search in past 6 months	No	621	55.0	29.85 ± 5.566	0.468	0.640
	Yes	509	45.0	29.69 ± 5.57		
Health app usage frequency	Never or rarely	559	49.5	28.53 ± 5.557	23.836	< 0.001
	Several times per month	313	27.7	30.28 ± 5.304		
	Several times per week	167	14.8	31.95 ± 4.934		
	Several times daily	91	8.1	31.74 ± 5.611		
Health information retrieval efficiency	Not at all	5	0.4	15.4 ± 10.854	76.265	< 0.001
	Not very	229	20.3	27.1 ± 5.583		
	Average	534	47.3	28.97 ± 4.904		
	Fairly capable	333	29.5	32.37 ± 4.543		
	Fully capable	29	2.6	38.48 ± 3.089		
Ability to distinguish authoritative sources	Not at all	13	1.2	22.31 ± 9.664	91.247	< 0.001
	Not very	164	14.5	26.15 ± 5.06		
	Moderate	397	35.1	28.2 ± 4.831		
	Fairly capable	464	41.1	31.39 ± 4.657		
	Fully capable	92	8.1	35.93 ± 4.484		
Medical knowledge judgment ability	Not at all	9	0.8	19 ± 9.354	72.285	< 0.001
	Not very	40	3.5	26.85 ± 6.407		
	Average	386	34.2	27.38 ± 5.105		
	Fairly capable	592	52.4	30.76 ± 4.75		
	Fully capable	103	9.1	35.16 ± 4.597		
Multi-channel verification behavior	Not at all	10	0.9	23.7 ± 11.392	50.419	< 0.001
	Not very	86	7.6	26.58 ± 5.842		
	Average	282	25.0	27.65 ± 5.34		
	Fairly capable	547	48.4	30.11 ± 4.675		
	Fully capable	205	18.1	33.43 ± 5.258		

Regarding health status and health-related digital use behaviors characteristics (Table 2), DHL scores were significantly associated with multiple variables in this domain. Specifically, students with better self-rated health, shorter daily internet usage duration, regular use of laptops/tablets and other digital devices, higher frequency of medical learning network usage, and prior systematic training in information retrieval or evidence-based medicine had significantly higher DHL scores (all *P* < 0.05). For example, significant differences were found in DHL scores based on the frequency of medical learning network usage (*F* = 13.375, *P* < 0.001) and prior information retrieval/evidence-based medicine training (*F* = 15.258, *P* < 0.001). No statistically significant differences in DHL scores were found regarding the presence of chronic diseases requiring long-term management, smartphone use, desktop computer use, or types of daily internet access (all *P* > 0.05).

For health information behaviors and competencies (Table 3), DHL scores were strongly associated with all core competency variables in this domain (all *P* < 0.001), except for the frequency of health information searches in the past 6 months (*P* = 0.640). Specifically, students with higher frequency of health-related app usage, higher self-rated efficiency in health information retrieval, stronger ability to distinguish authoritative sources, better medical knowledge judgment capacity, and proactive multi-channel verification behavior had significantly higher DHL scores (all *P* < 0.001). For instance, the most significant difference was observed in the ability to distinguish authoritative sources (*F* = 91.247, *P* < 0.001), followed by health information retrieval efficiency (*F* = 76.265, *P* < 0.001).

Regarding health information source utilization, which was analyzed separately in the text, search engines (78.5%), social media (64.7%), and online video platforms (62.7%) were the most commonly used channels among medical undergraduates. Peer communication (39.3%), school curriculum/teacher recommendations (32.3%), and professional medical databases (31.4%) were less frequently used, while only 11.4% of students reported using other sources. On average, respondents used 3.2 different health information sources in their daily lives.

Univariate analysis showed that students who used professional databases (31.46 ± 5.77 vs. 29.00 ± 5.30, *P* < 0.001), school curriculum/teacher recommendations (30.88 ± 5.45 vs. 29.25 ± 5.55, *P* < 0.001), and peer communication (30.42 ± 5.54 vs. 29.36 ± 5.55, *P* = 0.002) had significantly higher DHL scores. No significant differences in DHL scores were observed between users and non-users of search engines (*P* = 0.761), social media (*P* = 0.136), online video platforms (*P* = 0.004), or other sources (*P* = 0.579) after Bonferroni correction (adjusted α = 0.007).

### 3.4 Hierarchical multiple linear regression analysis of factors predictors DHL

Hierarchical multiple linear regression was conducted to identify independent predictors of DHL. Three models were constructed sequentially: Model I included only sociodemographic variables; Model II added health status and health-related digital use behaviors characteristics; Model III further incorporated health information behaviors and competencies.

The model fit improved significantly with each step: the adjusted *R*^2^ values for Models I, II, and III were 0.067, 0.123, and 0.353, respectively, and all model *F* tests were statistically significant at the *P* < 0.001 level (Table 4). This indicates that the behavioral determinants included in our study are the most important predictors of DHL among medical undergraduates. The remaining 64.7% of variance may be explained by unmeasured factors such as academic self-efficacy, learning motivation, personality traits, and the quality of university information literacy education, which should be further explored in future research.

**Table 4:** Hierarchical multiple linear regression analysis of factors influencing DHL.

Variable	Model I		Model II		Model III	
	β-value	*P*-value	β-value	*P*-value	β-value	*P*-value
Socio-demographic characteristics						
Gender (control = female)						
Male	0.769	0.021	0.519	0.116	0.189	0.518
Family residence (reference = urban)						
Township	−0.794	0.074	−0.669	0.127	−0.301	0.430
Rural	−0.224	0.622	−0.111	0.805	0.174	0.655
Ancestral hometown (reference = outside province)						
Within province	−0.738	0.167	−0.403	0.442	−0.206	0.651
Only child (control = non-only child)						
Only child	0.268	0.489	0.344	0.366	−0.029	0.931
Father's highest education level (reference = elementary school or below)						
Junior high school	1.514	0.016	1.305	0.034	0.858	0.109
High school/vocational school	1.957	0.005	1.785	0.009	1.071	0.072
College	1.504	0.075	1.229	0.138	0.546	0.451
Undergraduate	1.427	0.092	1.278	0.124	0.545	0.453
Graduate degree and above	1.698	0.201	1.316	0.315	0.478	0.674
Mother's highest education level (reference = elementary school or below)						
Junior high school	0.060	0.901	0.105	0.823	0.137	0.735
High school/vocational school	0.299	0.615	0.341	0.559	0.278	0.585
Junior college	0.604	0.439	0.539	0.481	0.333	0.620
Undergraduate	1.118	0.159	0.825	0.288	0.470	0.489
Graduate degree and above	0.562	0.694	0.733	0.600	0.488	0.688
Self-assessment of household financial situation (reference = very difficult)						
Somewhat difficult	−1.662	0.067	−1.291	0.147	−0.878	0.257
General	−0.539	0.529	−0.192	0.820	−0.179	0.808
Relatively comfortable	1.126	0.251	1.161	0.228	0.357	0.671
Very comfortable	1.732	0.413	1.889	0.361	0.783	0.664
Health Status and health-related digital use behaviors characteristics						
Daily internet usage duration (control = ≤ 2)						
2–4			−0.045	0.965	−0.763	0.396
4–6			−0.930	0.362	−1.252	0.157
6–8			−1.275	0.228	−1.391	0.130
>8			−0.927	0.393	−0.337	0.722
Frequency of medical learning network usage (control = rarely or never)						
Multiple times daily			3.209	< 0.001	1.562	0.003
Once daily			2.105	< 0.001	1.063	0.057
Several times per week			2.485	< 0.001	1.135	0.018
Several times a month			2.135	< 0.001	0.875	0.110
Frequently used digital devices (control = not used)						
Laptop/Tablet			0.448	0.219	−0.135	0.671
Other (smartwatches, etc.)			0.947	0.075	−0.141	0.766
Information retrieval/evidence-based medicine training (control = rarely or never)						
Systematically studied			1.570	< 0.001	0.495	0.244
Briefly familiar			0.661	0.062	0.184	0.556
Health information behavior and competency characteristics						
Health app usage frequency (control = never or rarely used)						
Several times per month					0.913	0.006
Several times per week					1.818	< 0.001
Several times daily					1.545	0.004
Health information retrieval efficiency (control = average)						
Not at all/not very					−0.996	0.010
Fairly/completely able					1.928	< 0.001
Ability to distinguish authoritative sources (control = average)						
Not at all/not very					−1.134	0.014
Somewhat/completely able					1.917	< 0.001
Medical knowledge judgment ability (control = average)						
Not at all/not very					−1.801	0.013
Somewhat/completely					1.108	0.001
Multi-channel verification behavior (control = average)						
Not at all/not very likely					0.108	0.852
Somewhat able/completely able					1.157	0.001
Professional databases (control = no)					0.876	0.007

In the final model, the following factors emerged as significant positive predictors of DHL: higher frequency of medical learning network usage (β = 1.562, *P* = 0.003), frequent use of health-related apps (several times per week: β = 1.818, *P* < 0.001; several times daily: β = 1.545, *P* = 0.004), greater efficiency in health information retrieval (β = 1.928, *P* < 0.001), stronger ability to distinguish authoritative sources (β = 1.917, *P* < 0.001), better medical knowledge judgment ability (β = 1.108, *P* = 0.001), proactive multi-channel verification behavior (β = 1.157, *P* = 0.001), and reliance on professional medical databases as a health information source (β = 0.876, *P* = 0.007). Conversely, deficits in information retrieval efficiency (β = −0.996, *P* = 0.010), ability to distinguish authoritative sources (β = −1.134, *P* = 0.014), and medical knowledge judgment ability (β = −1.801, *P* = 0.013) were significant negative predictors.

After adjusting for health-related digital use behaviors characteristics and health information competencies, no sociodemographic variables (including gender, place of origin, parental education, or family economic status) remained significantly associated with DHL (all *P* > 0.05).

## 4 Discussion

In this study, the total DHL score among undergraduate medical students in central China was 29.78 ± 5.57, indicating a moderate level. After adjusting for health-related digital use behaviors and information processing competencies, all sociodemographic variables were no longer significantly associated with DHL. Hierarchical regression analysis revealed that health information retrieval efficiency, ability to identify authoritative sources, medical knowledge judgment capacity, multi-channel verification behaviors, frequency of medical learning website usage, frequency of health-related app usage, and reliance on professional medical databases were independent positive correlates of DHL. These findings suggest that DHL among medical undergraduates is primarily associated with modifiable behavioral factors rather than innate demographic backgrounds, providing empirical evidence for universal competency-oriented training programs in medical education.

### 4.1 International comparison of DHL levels and regional characteristics

The DHL level observed in this study (29.78 ± 5.57) is broadly consistent with the pooled mean eHEALS score of medical students worldwide (28.6, 95% CI: 26.3–30.9) (8). Compared with domestic studies, our results are comparable to multiple previous studies conducted among Chinese medical students, all of which point to a consistent pattern of “adequate basic awareness but weak higher-order competencies” (11). Specifically, medical students generally possess the awareness to actively search for health information and basic operational skills, but exhibit notable deficiencies in advanced skills such as critical appraisal of health information and multi-source verification (12).

When compared with studies from other countries (38), the DHL score in our sample was significantly higher than that reported among university students in Pakistan (39) and Kenya (40), comparable to that of Tunisian medical students (28.22 ± 6.85) (41), but lower than the performance of Australian undergraduate health profession students (13). Previous cross-national comparisons have often attributed these differences solely to disparities in economic development levels. However, our findings suggest that this explanation may be overly simplistic. Australian universities have mandated digital health training as a core competency for all medical students and implemented a longitudinal curriculum spanning all 5 years (42, 43), while most medical schools in China and many developing countries only offer elective courses or occasional lectures on this topic (44). This contrast suggests that institutional factors—rather than individual characteristics or economic resources—may be important structural factors associated with population-level differences in DHL (13, 42).

### 4.2 The dominant role of behavioral factors and the “disappearance” of sociodemographic associations

The most central finding of this study is that after incorporating health-related digital use behaviors and information processing competencies, all sociodemographic variables were no longer significantly associated with DHL. This result contrasts with the conclusion of Estrela et al.'s (22) systematic review, which emphasized that DHL is associated with sociodemographic, economic, and cultural factors. However, this apparent discrepancy may reveal an important issue overlooked by previous research—studies that treated sociodemographic factors as primary predictors often failed to simultaneously control for behavioral variables, potentially overestimating the direct strength of association of sociodemographic factors while underestimating the mediating role of behavioral factors (23).

Our findings are consistent with a “behavioral mediation model” interpretation: sociodemographic characteristics are not directly associated with DHL levels but are indirectly associated with DHL through their influence on individuals' access to and engagement with digital health resources. In the Chinese medical education system, most medical schools provide all students with free access to major international and domestic medical databases through campus library subscriptions, combined with mandatory information literacy courses for all first-year students. This institutional support creates a relatively equitable starting point, which is associated with the observation that students from rural areas or low-income families can achieve DHL levels comparable to their more advantaged peers. This finding suggests that equitable institutional policies may be associated with the promotion of health equity, even in contexts with significant socioeconomic inequalities (12).

Notably, sociodemographic variables in this study all showed significant associations with DHL in univariate analyses but lost statistical significance in multivariate regression. This pattern is consistent with the mediation interpretation described above—the effects of sociodemographic factors are “explained” by behavioral factors, suggesting that the sociodemographic differences observed in previous literature may be closely related to behavioral differences in essence, rather than being independent correlates. This is consistent with the conclusions of Bautista et al.'s (23) systematic review among college students, which found that individuals' digital usage habits and information processing behaviors explain more variance in eHealth literacy levels than static demographic characteristics.

### 4.3 Educational implications of the absence of grade-level differences

A particularly noteworthy finding of this study is the absence of significant differences in DHL scores across academic years (first to fifth year, *F* = 0.872, *P* = 0.481). This result contrasts with the expectation that students' digital health information processing capabilities would naturally improve as they advance through medical training and gain more clinical exposure. The data indicate that even fourth- and fifth-year students, after years of medical coursework and clinical rotations, do not exhibit significantly higher DHL levels than their junior counterparts.

This finding contrasts with studies conducted in Turkey and Australia, where medical students' DHL scores showed a steady upward trend with each academic year—a pattern directly attributed to longitudinally integrated digital health curricula (13, 46, 47). The Turkish study demonstrated significant differences in eHEALS scores across year levels, with senior students performing better (46); the Australian study showed that systematic digital health skills training effectively improves students' eHealth literacy (13, 47).

Three key systemic factors in the current Chinese medical education system may be associated with this pattern of developmental stagnation. First, digital health literacy training is almost exclusively delivered as a single, isolated 16-h information literacy module in the first year, with no subsequent reinforcement or advanced training in later years (12). Second, clinical rotations—the most critical period for translating theoretical knowledge into practical competence—rarely include explicit digital health training. While students frequently use smartphones and online resources to support their clinical work, they receive no formal guidance from attending physicians on how to evaluate the quality of online clinical guidelines or apply digital health tools in patient care (14, 15). Third, the digital skills training provided does not align with the evolving needs of students at different training stages—the same basic keyword search skills taught to first-year students are not expanded to meet the advanced clinical information needs of fourth- and fifth-year students, who require skills in appraising systematic reviews, clinical practice guidelines, and real-world evidence (12, 48). Notably, this pattern emerges despite universal free access to comprehensive international and domestic medical databases through the university library, further suggesting that resource availability alone may be insufficient to improve DHL without structured, progressive training (12).

This finding prompts a re-examination of how DHL develops in medical education: DHL may not be a passive byproduct of general medical training, but rather a specific competency that requires intentional, longitudinal cultivation throughout the undergraduate curriculum. This carries clear implications for medical education reform—medical schools may benefit from adopting a longitudinal, spiral curriculum model for digital health literacy, with targeted content at different year levels to enable students' DHL to develop in tandem with their clinical competence (42, 47).

### 4.4 Theoretical contributions and methodological reflections

This study makes three theoretical contributions to the field of digital health literacy research. First, it offers a complementary perspective to the “structural determinism” view—which posits that DHL disparities are primarily associated with socioeconomic inequalities (22)—by providing empirical support for a “behavioral mediation model.” This shift in perspective has implications for how we conceptualize DHL disparities and design interventions to address them.

Second, this study provides empirical evidence suggesting that DHL is an acquirable skill. In the Chinese medical education system, universal free access to major international and domestic medical databases through campus library subscriptions, combined with mandatory information literacy courses for all first-year students, is associated with mitigating the relationship between socioeconomic disadvantage and DHL development. This institutional support creates a relatively equitable environment, which is associated with the observation that students from rural or low-income backgrounds can achieve DHL levels comparable to their more advantaged peers (12).

Third, this study further supports the conceptual distinction between perceived DHL (measured by eHEALS) and actual health information processing behaviors. Consistent with the findings of Neter and Brainin—who reported that perceived eHealth literacy and actual performance are only moderately correlated (*r* = 0.34) (35)—our study also suggests that while these two constructs are correlated, they may measure different aspects of digital health competence: eHEALS assesses perceived self-efficacy, while our behavioral measures focus more on self-reported actual behaviors and context-specific capabilities (35, 45). This distinction is important because many previous studies have relied solely on self-reported DHL measures, which may overestimate actual competence in critical evaluation skills (45).

### 4.5 Practical implications

The above findings provide evidence-informed directions for medical education reform. First, medical schools may shift their focus from targeted interventions for specific sociodemographic subgroups to universal competency-based training for all students. Given that sociodemographic factors are not independently associated with DHL, targeted interventions may have limited effectiveness in reducing disparities; instead, universal training focused on improving behavioral skills may benefit all students equally (9, 13).

Second, DHL training should be integrated longitudinally into the medical curriculum rather than offered as isolated one-off courses. The absence of significant grade-level differences in DHL scores is one of the most educationally meaningful findings of this study, as it suggests that students' DHL does not show significant improvement throughout their 5-year undergraduate training despite increasing clinical exposure and medical knowledge accumulation. To address this, medical schools may consider adopting a longitudinal, spiral curriculum model for digital health literacy: basic digital navigation and database search skills could be introduced in the first year; critical appraisal of research articles and evidence-based medicine skills could be reinforced in the second and third years; and advanced skills such as clinical decision support tools, patient digital health education, and misinformation identification could be integrated into clinical rotations in the fourth and fifth years (42, 47).

Third, training programs should prioritize higher-order skills such as critical evaluation of health information and multi-source verification. Our study found that these skills are the strongest correlates of DHL, yet they are among the most deficient in medical students (11, 12). Specifically, training could include: (1) hands-on workshops on advanced search strategies in PubMed, Cochrane Library, and Chinese biomedical databases; (2) case-based exercises in identifying common types of health misinformation; and (3) role-playing activities where students practice explaining evidence-based health information to patients with limited health literacy (13, 14).

### 4.6 Limitations and future directions

This study has several limitations. First, the cross-sectional design precludes causal inferences—all associations identified in this study should be interpreted as correlational rather than causal. Second, the eHEALS measures self-perceived abilities, which may be subject to social desirability bias (35). Third, we adopted stratified cluster sampling with classes as clusters, but cluster effect adjustment was not applied in all inferential statistical analyses; this methodological limitation may affect the precision of effect estimates. Fourth, some predictor variables in the regression model have inherent conceptual proximity to the domains measured by the eHEALS scale. Although we have clarified the theoretical and measurement distinctions between perceived self-efficacy and actual practical capabilities, this conceptual overlap may have influenced the strength of statistical associations observed in this study (45). Therefore, results concerning the associations between behavioral factors and DHL—particularly those involving conceptually proximate variables—should be interpreted with caution.

Future research should adopt multi-center longitudinal designs to further explore the direction of associations between behavioral factors and DHL development. Additionally, future studies could incorporate objective performance-based assessments (e.g., simulated health information search tasks) to complement self-reported measures and more comprehensively evaluate actual digital health competence (35). Finally, cluster-randomized controlled trials could be conducted to evaluate the effectiveness of the longitudinal, spiral curriculum model proposed in this study.

## 5 Conclusion

This study demonstrates that DHL among Chinese medical undergraduates is primarily predicted by modifiable individual factors, including health information retrieval efficiency, critical information evaluation ability, and standardized use of authoritative digital resources, rather than innate sociodemographic background. The most critical finding of this study is that sociodemographic characteristics lose all predictive significance for DHL after accounting for digital practice behaviors and core information competencies, and its most important theoretical contribution is empirically supporting the core view that “DHL is an acquirable skill, not a privilege associated with sociodemographic background at birth.” This conclusion indicates that medical schools should prioritize universal, competency-based standardized training over targeted interventions for specific demographic subgroups. Specifically, curricula should integrate specialized DHL training modules into existing compulsory courses, focus on cultivating students' ability to critically evaluate digital health information, promote the standardized application of professional medical databases, and implement equal-access standardized skills training for all students. Future multi-center longitudinal studies with objective performance-based assessment tools are needed to validate these findings and develop more evidence-based educational intervention strategies for medical undergraduates.

## Data Availability

The original contributions presented in the study are included in the article/Supplementary material, further inquiries can be directed to the corresponding author.
