# Supplementary material for: Digital health literacy and its behavioral correlates among medical undergraduates in central China: a cross-sectional study
Source: Front Public Health. 2026 Jul 9;14:1880430. doi: 10.3389/fpubh.2026.1880430 (PMC13391500; doi:10.3389/fpubh.2026.1880430)
Supplement: Supplementary file 1 [file Data_Sheet_1.pdf]

Supplementary Material S1: Questionnaire

Research Topic: A Cross-sectional Study on Digital Health Literacy and Its Behavioral Determinants Among Medical Undergraduates in Anhui Province

Ethics Approval Number: SL-YX2026-097

Survey Period: October–December 2025

Dear Student: Greetings! We are conducting academic research on the digital health literacy of medical undergraduates. This questionnaire is anonymous, and all collected data will be used solely for academic analysis; no personal information will be disclosed. Completing the questionnaire will take approximately 8–10 minutes.

Your participation is entirely voluntary. You may withdraw at any time during the process without any adverse consequences. By continuing to complete and submit this questionnaire, you voluntarily consent to participate in this study. Thank you for your support and cooperation!

Part Ⅰ: Demographic and Sociological Characteristics

Unless otherwise noted, all questions are single-choice

1. Gender:

□ Male □ Female

2. Age:

□ 18 years old or younger □ 19 years old □ 20 years old or older

3. Place of Origin:

□ Urban □ Suburban □ Rural

4. Place of Origin:

☐ Within Anhui Province ☐ Outside Anhui Province

5. Major:

☐ Clinical Medicine □ Medical Technology □ Public Health and Preventive Medicine
□ Health Management □ Other

6. Year of Study:

□ Freshman □ Sophomore □ Junior □ Senior □ Fifth Year

(Note on Data Analysis: For statistical purposes, groups are combined; lower-year students = Freshmen through Juniors; upper-year students = Seniors through Fifth-Year Students)

7. Have you received a scholarship?

□ Yes □ No

8. Are you an only child?

□ Yes □ No

9. Father's Highest Level of Education:

□ Elementary School or Below □ Junior High School □ High School / Vocational School
□ Associate's Degree □ Bachelor's Degree □ Master's Degree or Higher

10. Mother's highest level of education:

□ Elementary school or below □ Junior high school □ High school / vocational school
□ Associate's degree □ Bachelor's degree □ Master's degree or higher

11. Self-assessment of family financial situation:

□ Very difficult □ Somewhat difficult □ Average □ Somewhat comfortable □ Very comfortable

Part Ⅱ: Health Status and Digital Usage Behaviors

1. Overall self-assessed health:

□ Very poor □ Fairly poor □ Average □ Fairly good □ Very good

2. Do you have a chronic condition requiring long-term management?
□ Yes □ No
3. Average Daily Internet Usage:
□ Less than 2 hours □ 2–4 hours □ 4–6 hours □ 6–8 hours □ More than 8 hours
4. Digital devices used daily (multiple selections allowed):
□ Smartphone □ Laptop / Tablet □ Desktop Computer □ Other (e.g., smartwatch, etc.)
(Data Analysis Note: Each option is treated as a separate binary variable; "Use" = 1, "Do not use" = 0)
5. Types of internet used daily (multiple selections allowed):
□ Campus Wi-Fi □ Mobile data □ Public Wi-Fi
(Data analysis note: Each option is treated as a separate binary variable; use = 1 if selected, = 0 if not)
6. Monthly mobile data usage:
□ 10 GB or less □ 11–30 GB □ 31 GB or more
7. Frequency of using the internet for medical studies:
□ Multiple times a day □ Once a day □ Several times a week □ Several times a month □ Rarely or never
8. Have you received training in information retrieval or evidence-based medicine?
□ Systematic training □ Basic understanding □ Never received training
9. Have you searched for health-related information online in the past 6 months:
□ Yes □ No
10. Primary channels for obtaining health information (multiple selections allowed):
□ Professional medical databases □ Search engines □ Social media □ Online video platforms
□ School courses / Teacher recommendations □ Peer discussions □ Other
(Data analysis notes: Each option is treated as a separate binary variable; Bonferroni correction is applied for multiple comparisons, with a corrected significance level of α=0.007)
11. Frequency of using health-related apps in the past 6 months:
□ Never / Rarely □ Several times a month □ Several times a week □ Several times a day

Part Ⅲ: Health Information Processing Ability Scale

***Instructions:**Please rate each statement on a 5-point scale (1 = Not at all, 2 = Not very much, 3 = Somewhat, 4 = Quite a bit, 5 = Very much).*

Item	1	2	3	4	5
1.I am able to quickly retrieve the health information I need.	□	□	□	□	□
2.I can distinguish between authoritative and non-authoritative sources of health information.	□	□	□	□	□
3.I can determine whether health information aligns with medical common sense.	□	□	□	□	□
4.I cross-verify the accuracy of health information through multiple channels.	□	□	□	□	□

Part IV: Chinese Version of the eHealth Literacy Scale (eHEALS)

Scale Description: This scale was developed by Norman and Skinner and adapted into Chinese *Source: Norman & Skinner (2006); Chinese adaptation: Guo et al. (2013) . Instructions: Please

rate each statement on a 5-point scale (1 = Strongly Disagree, 2 = Disagree, 3 = Neutral, 4 = Agree, 5 = Strongly Agree).*

Item	1	2	3	4	5
1.I know how to use online health resources to help myself.	□	□	□	□	□
2.I know where to find useful health resources.	□	□	□	□	□
3.I know how to use the internet to answer health-related questions.	□	□	□	□	□
4.I know how to evaluate the quality of online health resources.	□	□	□	□	□
5.I can efficiently access health resources online.	□	□	□	□	□
6.I am confident in making health-related decisions based on online information.	□	□	□	□	□
7.I can distinguish between high-quality and low-quality health resources online.	□	□	□	□	□
8.I know how to use online health resources to address my own health issues.	□	□	□	□	□
Supplementary item 1: The internet is very important to me when making health decisions.	□	□	□	□	□
Supplementary item 2: I need help from others when accessing online health information.	□	□	□	□	□

Note: Items 1–8 are scored for the total eHEALS score (range: 8–40). Supplementary items are for exploratory analysis only and are not included in the total score calculation.

Survey Closing Remarks

This concludes the survey. Thank you again for your careful completion and cooperation!
